# Preemptive middle glenohumeral ligament release in arthroscopic rotator cuff repair does not reduce the postoperative stiffness: a retrospective comparative study

**DOI:** 10.1186/s12891-023-06611-7

**Published:** 2023-06-15

**Authors:** Ryosuke Takahashi, Yukihiro Kajita, Yohei Harada, Yusuke Iwahori

**Affiliations:** 1 Department of Orthopaedic Surgery, Ichinomiya Nishi Hospital, 1Jihira, Ichinomiya City, 494-0001 Kaimei, Aichi Japan; 2 Department of Orthopaedic Surgery, Graduate School of Biomedical and Health Sciences, Hiroshima University, 1-2-3 Kasumi, Minami-ku, Hiroshima City, 734-8551 Hiroshima Japan; 3 Sports Medicine and Joint Center, Asahi Hospital, 2090 Shimoharacho Azamurahigashi, Kasugai, 486-0819 Aichi Japan

**Keywords:** Preemptive middle glenohumeral ligament release, Postoperative stiffness, Arthroscopic rotator cuff repair

## Abstract

**Background:**

This study aimed to evaluate the efficacy of preemptive middle glenohumeral ligament (MGHL) release in arthroscopic rotator cuff repair (ARCR) to reduce postoperative stiffness.

**Methods:**

Patients who underwent ARCR were enrolled and allocated into two groups retrospectively: the preemptive MGHL release group (n = 44) and the preemptive MGHL non-release group (n = 42). Clinical outcomes were assessed and compared between the two groups, including the range of motion, Japanese Orthopedic Association Shoulder Score, Constant Shoulder Score, and the University of California, Los Angeles Score preoperatively and 3 months, 6 months, and 12 months postoperatively and complications. The integrity of the repaired tendon was assessed at the 12-month follow-up using magnetic resonance imaging.

**Results:**

There were no significant differences between the groups in all range of motion and all functional scores at any of the assessed time points. There was also no significant difference in the healing failure rate 2.3% in the preemptive MGHL group and 2.4% in the preemptive MGHL non-release group (p = .97), and postoperative stiffness was 2.3% in the preemptive MGHL group and 7.1% in the preemptive MGHL non-release group (p = .28). There was no postoperative instability in both group.

**Conclusion:**

ARCR effectively facilitates the recovery of range of motion and function in patients with a rotator cuff tear. However, preemptive MGHL release could not be an effective method to reduce postoperative stiffness.

## Introduction

Although arthroscopic rotator cuff repair (ARCR) is a minimally invasive procedure, postoperative stiffness may still develop and lead to less functional outcomes [1–3]. Surgeons have made several efforts to prevent postoperative stiffness, for instance, encouraging early passive shoulder exercise [4], injecting an anti-adhesive agent into the subacromial space or glenohumeral joint postoperatively [5], or the combination of ARCR with either manipulation under anesthesia or arthroscopic capsular release [6, 7], however, the efforts remain controversial and lack of consensus [6, 8–12].

The main causes of shoulder stiffness have been reported to be the thickening of the coracohumeral ligament (CHL) and joint capsule in the rotator interval (RI) or obliteration of the fat triangle between the coracoid process and the CHL [11–13]. Postoperative stiffness has a greater component of intra-articular causes, predominantly capsular fibrosis and adhesions arising from the bodily reactions to the damaged glenohumeral ligaments [3, 14, 15]. Nevertheless, there have been few studies on intraoperative surgical procedures to reduce postoperative stiffness after ARCR in patients with no preoperative stiffness [11, 12].

Although the RI capsule containing the CHL is considered the predominant area of the stiffed shoulder [11–13], it is difficult to gain a full range of motion (ROM) after the release of only the RI capsule [16]. Holloway et al. reported that wide arthroscopic capsule release is necessary for regaining the full ROM in a stiffed shoulder, which indicates that the capsule, including the glenohumeral ligaments, is one of the main causes of the restricted ROM [17].

The middle glenohumeral ligament (MGHL) is one of the three ligaments that reinforce the anterior glenohumeral capsule along with the superior (SGHL) and the inferior (IGHL) glenohumeral ligaments [18, 19], which respectively connect the anterosuperior labrum to the top of the bicipital groove, and the inferior part of the glenoid to the inferomedial aspect of the surgical neck of the humerus. Many biomechanical studies emphasized the effect of MGHL on the anterosuperior stability of the shoulder [19–21]. Although thickening of the CHL that covers the RI is recognized as a causative factor limiting external rotation (ER) of the shoulder joint [12], and previous studies have reported the effect of the CHL release [22–25], it has been reported that the capsule, including the glenohumeral ligaments, is one of the main causes of a restricted ROM [17]. However, to our knowledge, there has been only one study on whether preemptive MGHL release as an intraoperative procedure would reduce postoperative stiffness [11]. Therefore, this study aimed to evaluate the efficacy and safety of MGHL release in ARCR to reduce postoperative stiffness. We hypothesized that patients who underwent ARCR with preemptive MGHL release would experience reduced postoperative stiffness than patients who underwent ARCR without MGHL release.

## Materials and methods

### Inclusion and exclusion criteria

**Fig. 1:**
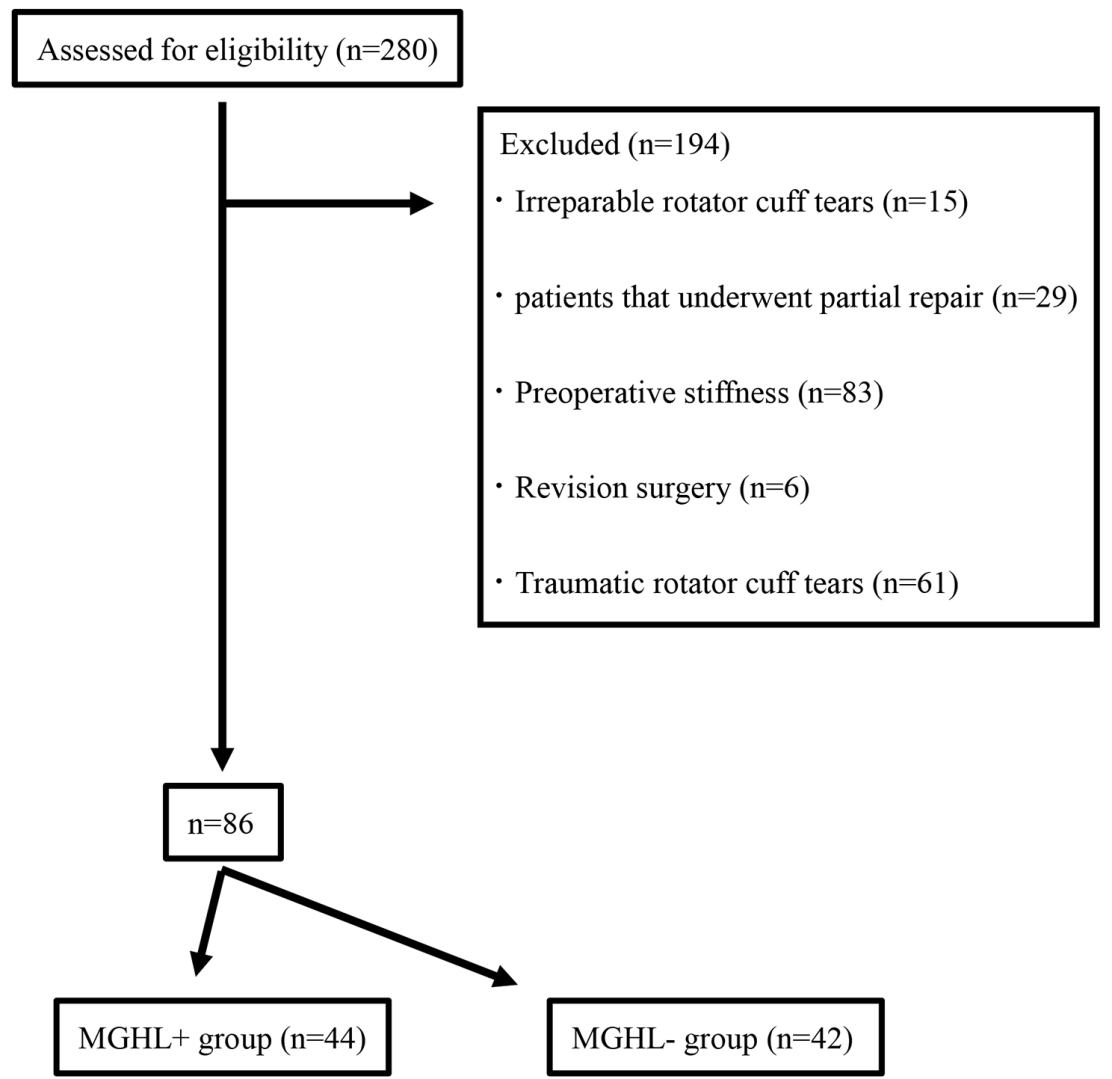
Study design flow diagram

Between January 2018 and May 2021, 280 consecutive patients underwent ARCR at our hospital. Informed consent was obtained from all participants, and institutional review board approved this study (2,023,008). We enrolled patients who met the following inclusion criteria: presence of complete rotator cuff tears, including the supraspinatus tendon as verified by preoperative magnetic resonance imaging (MRI); patients who underwent complete rotator cuff repair; and follow-up for at least one year after ARCR with an evaluation of successful repair using MRI. The exclusion criteria were as follows: irreparable rotator cuff tears, patients who underwent partial repair, preoperative shoulder stiffness, revision surgery and traumatic rotator cuff tears. Patients were divided into two groups: ARCR without MGHL release from January 2018 to December 2019 (MGHL- group) and ARCR with MGHL release from January 2020 to May 2021 (MGHL + group). The tear size of the rotator cuff was evaluated using MRI. We measured the longitudinal and transverse dimensions of the tear on preoperative MRI along the oblique coronal and sagittal planes, respectively [26]. The tear size was categorized as small (< 1 cm), medium (1–3 cm), large (3–5 cm), or massive (> 5 cm), according to Cofield [27]. We defined shoulder stiffness as limited shoulder ROM (passive forward flexion less than or equal to 120°and/or ER less than or equal to 30°), as previously described [28]. Patients who met these criteria were considered to have preoperative stiffness. A total of 280 ARCRs were performed during the study period. After the exclusion of 194 patients, the remaining 86 patients were included in this study (Fig. 1).

### Surgical technique

**Fig. 2:**
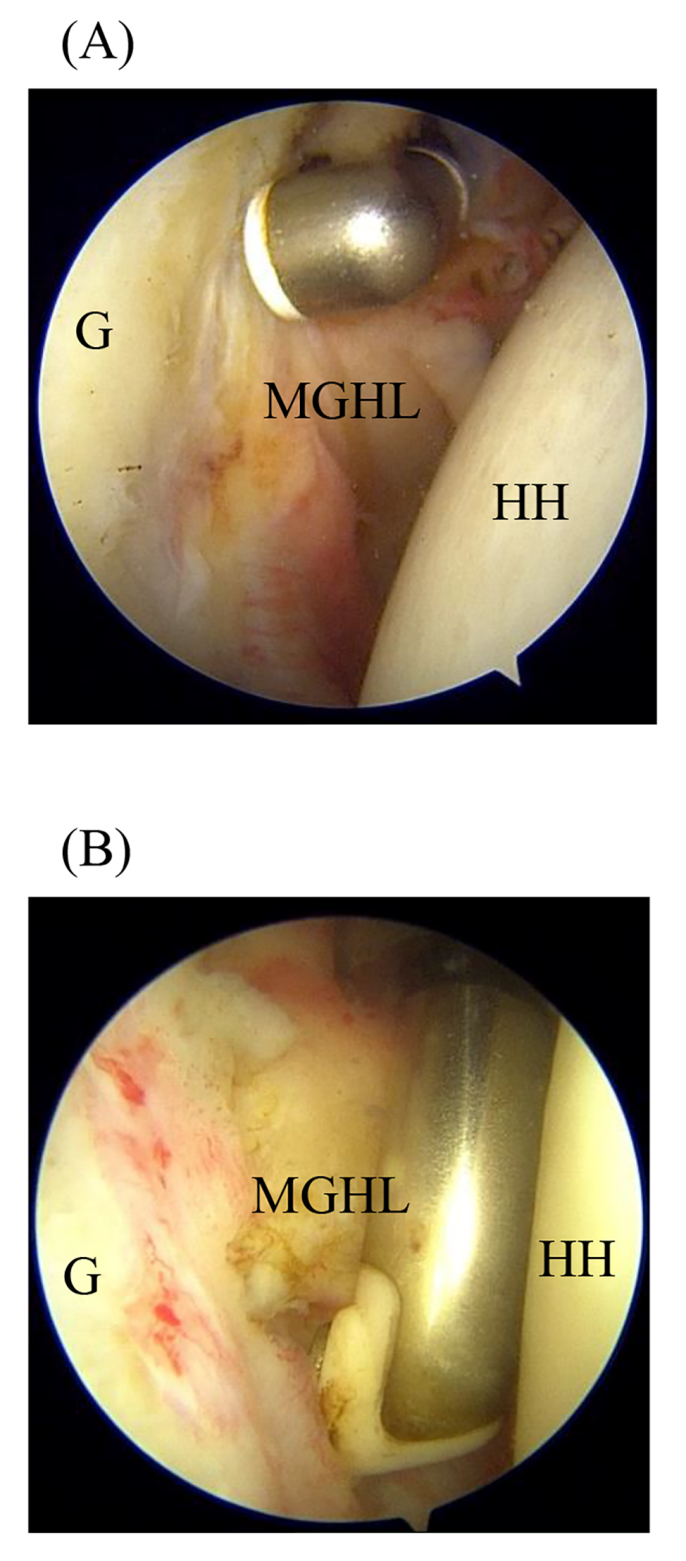
The MGHL release in the glenohumeral joint of the right shoulder **(A)** The radiofrequency device was inserted through the anterior portal into the glenohumeral joint (**B)** MGHL was released along the margin of the glenoid with the radiofrequency device. G; glenoid, HH; humeral head, MGHL; middle glenohumeral ligament

**Fig. 3:**
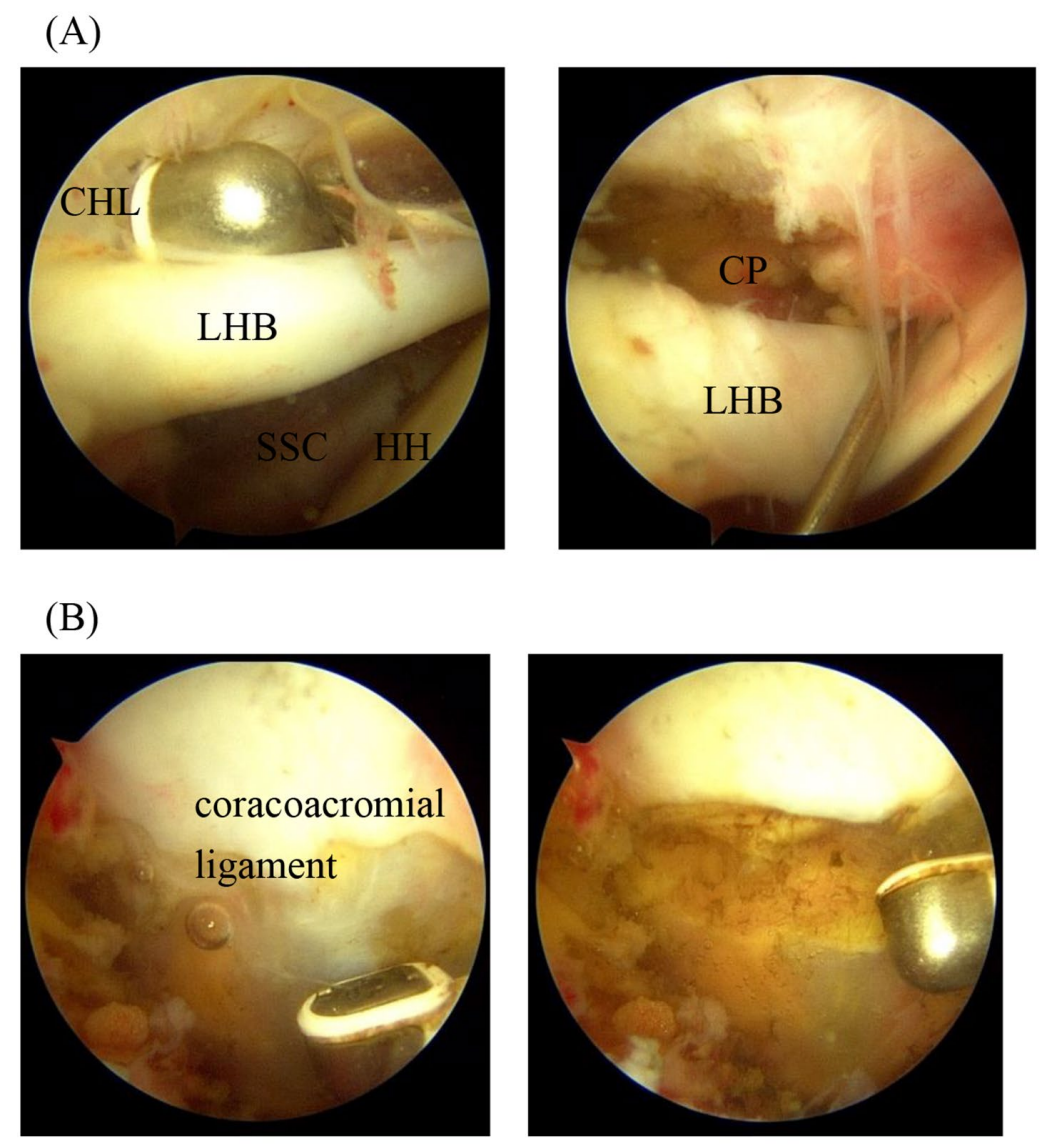
The CHL release and coracoacromial ligament release of the right shoulder. **(A)** The radiofrequency device was inserted through the anterior portal into the glenohumeral joint. The CHL was released until the base of the CP using a radiofrequency device. **(B)** The coracoacromial ligament was released into the subacromial space using a radiofrequency device. HH; humeral head, CHL; coracohumeral ligament, LHB; long head of biceps, SSC; subscapularis, CP; coracoid process

All operations were performed uniformly under general anesthesia and in a beach chair position by a single skilled surgeon. The arthroscope was inserted through the posterior portal, and a standard anterior portal was made a working portal in the RI capsule. After visualization, all hypertrophic synovial tissues were cleared as needed. In the MGHL + group, MGHL was released from the undersurface of the glenoid using a radiofrequency device through the anterior portal (Fig. 2). Then, the CHL release was performed until the base of the coracoid process into the glenohumeral joint using a radiofrequency device and the coracoacromial ligament was exposed into the subacromial space using a radiofrequency device in both groups (Fig. 3). Following the removal of the subacromial bursal tissue and bone spur, a standard ARCR was performed using suture anchors. The number of anchors was decided according to the size of the tear and repair configuration in the suture-bridge repair. In patients who also required the subscapularis tendon repair, the subscapularis tendon was repaired using the suture anchor by a single row technique. Tenotomy or tenodesis was performed in case of a biceps long head lesion.

All patients received the same postoperative rehabilitation [29]. The shoulder was immobilized for four weeks for small-to-medium tears and six weeks for large-to-massive tears using an abduction brace (Global Sling; COSMOS, Sapporo, Japan). The elbow, wrist, and fingers exercises were started immediately after surgery. Passive forward flexion exercises were initiated from the day after surgery. An active-assisted motion exercise was initiated at four weeks for small-to-medium tears and six weeks for large-to-massive tears postoperatively. An active motion was allowed at six weeks for small-to-medium tears and eight weeks for large-to-massive tears postoperatively. A strengthening exercise program was allowed at eight weeks for small-to-medium tears and 12 weeks for large-to-massive tears postoperatively. Rehabilitation was performed at least three months after surgery with the assistance of a physical therapist. Full return to sports or heavy labor was allowed after six months.

### Clinical outcomes

Clinical outcomes were evaluated between the two groups, including the ROM, Japanese Orthopedic Association (JOA) Shoulder Score, Constant Shoulder Score, and the University of California, Los Angeles (UCLA) Score preoperatively and 3 months, 6 months, and 12 months postoperatively and complications. Active ROM (forward flexion, ER, and internal rotation [IR]) were measured with the scapular in a fixed position. IR was defined as the highest vertebral body the patient could reach with the thumb of the affected arm. IR was scored in accordance with the JOA shoulder score as follows: above Th12, 6 points; above L5, 4 points; at the buttocks, 2 points; and below the buttocks, 0 points. The integrity of the repaired tendon was assessed at the 12-month follow-up using MRI. Repair integrity after ARCR was classified into five categories according to the Sugaya classification using oblique coronal, oblique sagittal, and transverse views of T2-weighted images [30]. Types 4 and 5 were considered retears using this classification system.

### Statistical analysis

All statistical analyses were performed using the SPSS software (ver.18.0, SPSS Inc, Chicago, IL, USA). The sample size for this study was set as the maximum number of cases that could be obtained during the study period. Therefore, post-hoc power analysis was performed on the actual sample size to calculate the power at moderate effect size (d = 0.5 [unpaired t-test], w = 0.3 [chi-square test]) and at large effect size (d = 0.8 [unpaired t-test], w = 0.5 [chi-square test]). The chi-squared test was used to analyze categorical variables to compare patients’ gender ratio, affected side, tear size, the number of rotator cuff tears, the procedure of biceps long head, the presence of diabetes mellitus, and complications between two groups. Student’s t-test was used to compare age, ROM, and functional scores between the two groups, and the paired t-test was used to compare these variables between two consecutive periods in each group. Statistical significance was set at p < .05.

## Results

A total of 280 ARCRs were performed during the study period. After excluding 194 patients, the remaining 86 were included in this study. The mean age of all patients was 62.2 ± 9.4 years and the mean follow-up period was 15.6 ± 3.5 months. Forty-four patients underwent ARCR with MGHL release (MGHL + group; 32 males and 12 females, mean age was 62.8 ± 8.9 years), and 42 underwent ARCR without MGHL release (MGHL- group; 23 males and 19 females, mean age was 61.7 ± 9.9 years).

The demographic characteristics of the patients are presented in Table 1. There were no significant differences in gender, mean age, affected side, tear size, the number of rotator cuff tears, the procedure of biceps long head, the presence of diabetes mellitus, ROM, and functional scores between the two groups. There were no significant differences between the groups in all ROM and all functional scores at any of the assessed time points (Tables 2 and 3). There was also no significant difference in the healing failure rate 2.3% in the MGHL + group and 2.4% in the MGHL- group (one patient in the large-sized tear in each group) (p = .97), and postoperative stiffness was one patient in the large-sized tear (2.3%) in the MGHL + group and 3 patients (two patients in the large-sized and one in the medium-sized tear) (7.1%) in the MGHL- group (p = .28) There was no postoperative instability in both group. (Table 4).

**Table 1:** Patient’s demographics at baseline

Variables	MGHL + group	MGHL- group	P-value
Number of shoulders	44	42	
Male / Female (n)	32/12	23/19	0.08
Age (years)	62.8 ± 8.9	61.7 ± 9.9	0.28
Affected side (right /left) (n)	29/15	21/21	0.14
Tear size (n)			0.95
small	28	30	
medium	13	9	
large	3	3	
massive	0	0	
The number of rotator cuff tear (n)			0.39
SSP only	36	31	
SSP + ISP	1	1	
SSP + SSC	6	8	
SSP + SSC + ISP	1	2	
Procedure of biceps long head (n)			0.27
tenotomy	21	20	
tenodesis	5	4	
nothing	18	16	
The presence of diabetes mellitus (n)	8	13	0.17

SSP: Supraspinatus

ISP: Infraspinatus

SSC: Subscapularis

**Table 2:** Comparison of preoperative and postoperative ROM

Variables	MGHL + group	MGHL- group	P-value
ROM			
forward flexion (°)			
Preop	157.9 ± 12.6	160.7 ± 15.6	0.18
POD 3 M	139.5 ± 20.3	142.1 ± 20.4	0.27
POD 6 M	160.5 ± 17.4	162.1 ± 13.3	0.32
POD 12 M	167.2 ± 11.2	169.2 ± 10.4	0.19
ER (°)			
Preop	52.9 ± 8.5	55.4 ± 6.7	0.07
POD 3 M	37.3 ± 10.1	40.2 ± 13.6	0.14
POD 6 M	50.3 ± 8.8	52.9 ± 11.1	0.11
POD 12 M	55.3 ± 7.4	56.9 ± 8.3	0.18
IR (point)			
Preop	4.0 ± 1.8	4.2 ± 1.7	0.31
POD 3 M	3.3 ± 1.5	3.2 ± 1.5	0.43
POD 6 M	4.5 ± 1.4	4.6 ± 1.2	0.33
POD 12 M	5.5 ± 0.9	5.6 ± 0.8	0.18

**Table 3:** Comparison of preoperative and postoperative functional scores

Variables	MGHL + group	MGHL- group	P-value
JOA score (point)			
Preop	64.5 ± 11.9	68.3 ± 12.5	0.08
POD 3 M	78.1 ± 5.6	80.1 ± 6.1	0.07
POD 6 M	86 ± 8.1	88.2 ± 7.8	0.09
POD 12 M	94 ± 5.3	94.2 ± 8.1	0.42
Constant Shoulder score (point)			
Preop	61.1 ± 11	63.1 ± 11.9	0.25
POD 3 M	78.1 ± 5.6	80.1 ± 6.1	0.26
POD 6 M	80.6 ± 8.1	83 ± 7.8	0.08
POD 12 M	89.5 ± 5.9	89.4 ± 8.3	0.46
UCLA score (point)			
Preop	18.5 ± 3.7	19.8 ± 4.7	0.16
POD 3 M	22.6 ± 2.5	21.9 ± 2.7	0.10
POD 6 M	26.3 ± 3.5	26.4 ± 3.5	0.44
POD 12 M	30.7 ± 3.2	29.8 ± 3.6	0.11

**Table 4:** Complications

	MGHL + group	MGHL- group	P-value
Re-tear	1 (2.3%)	1 (2.4%)	0.97
Postoperative stiffness	1 (2.3%)	3 (7.1%)	0.28
Postoperative instability	0	0	1.0

When post-hoc power analysis was performed on the actual sample size obtained, the power of unpaired t-test was 0.630 and chi-square test was 0.794 for a moderate effect size, while the power of unpaired t-test was 0.956 and chi-square test was 0.996 for a large effect size.

## Discussion

The main finding of this study was that the MGHL + group did not experience more reduced postoperative stiffness than the MGHL- group. Postoperative shoulder stiffness is a prevalent adverse event after ARCR that is associated with major limitations in daily activities and prolonged rehabilitaion [9, 31, 32].

The incidence of postoperative stiffness after ARCR has been reported to range from 4.91 to 32.7% and, if left untreated, may lead to substantial morbidity [31, 33, 34]. The exact etiology of postoperative stiffness has not been established yet; capsular contractures and postsurgical adhesion to the surrounding soft tissues are considered responsible for causing postoperative stiffness [32]. Preoperative risk factors for developing postoperative shoulder stiffness after ARCR have been reported to be preoperative shoulder stiffness, age less than 50 years, workers compensation, diabetes, hypothyroidism, and coexisting diagnosis of calcific tendonitis or adhesive capsulitis [9, 31, 35]. Intraoperative risk factors reported include single-tendon tears, partial articular-sided tears, and concomitant labral repair [31]. Several studies have reported that associated procedures, including long head of biceps tenotomy or tenodesis, acromioplasty, capsulotomy, and glenohumeral/acromioclavicular osteoarthritis, could also increase the rate of postoperative shoulder stiffness [36–38].

The CHL has been reported to originate from the base and horizontal limb of the coracoid process, enclosing the subscapularis, supraspinatus, and infraspinatus tendons [39]. In this study, it enveloped vaster areas of the subscapularis than previously reported [22]. A thickened CHL at the RI has been well known to be one of the most specific manifestations of a stiff shoulder and the primary restraint against ER [23]. Neer et al. reported that ER could be increased up to an average of 32° when sectioning CHL [24]. Harryman et al. reported that the sectioning of the RI increased the ROM of the shoulder [25]. Tsai et al. reported that arthroscopic extended RI release for patients with refractory adhesive capsulitis improved the shoulder ROM [40]. Furthermore, Jazrawi et al. examined the effects of arthroscopic RI closure and found that imbrication of the RI resulted in a loss of approximately 11° of ER [41]. Mologne et al. also reported that arthroscopic RI closure significantly reduced ER in both neutral and abducted arm positions [42]. These studies suggested that the RI is closely associated with the ROM of the shoulder.

If postoperative stiffness is not resolved, additional procedures such as manipulation under anesthesia or arthroscopic capsular release could be considered [6, 7, 10]. Although many trials have been conducted on these clinical factors, only a few studies have investigated intraoperative procedures to prevent postoperative stiffness [11, 12]. Kim et al. reported that preemptive RI release in ARCR presented significantly better ROM and functional scores at postoperative 3 months than in the RI non-release group [11]. However, the functional scores and ROM were not significantly different between the two groups at postoperative 6 or 12 months or the final follow-up. Park et al. reported that concomitant CHL release in ARCR presented significantly better ER in the early postoperative period than in the CHL non-release group, which was effective in patients with a small-to-medium-sized rotator cuff tear [12]. They concluded that CHL release in ARCR can be used as a selective procedure to prevent postoperative stiffness in patients that may benefit from this procedure with decreased preoperative ER compared to the normal side.

The MGHL is one of the three ligaments that reinforce the anterior glenohumeral capsule along with the SGHL and IGHL [18, 19]. It originates from the anterior margin of the glenoid and crosses the subscapularis tendon during its course and attaches inferior of the SGHL attachment side [43]. Many biomechanical studies emphasized the effect of MGHL on the anterosuperior stability of the shoulder [19–21].The MGHL is one of the first anatomical elements observed during arthroscopic explorations of the glenohumeral joint from a posterior approach. As previously mentioned, although thickened CHL at the RI has been well known to be one of the most specific manifestations of a stiff shoulder [23], it has been reported that the capsule, including the glenohumeral ligaments, is one of the main causes of a restricted ROM [17]. Therefore, we considered that the MGHL, which is adjacent to CHL and RI, is also one of the causes of stiff shoulder and we hypothesized that preemptive MGHL release could prevent postoperative stiffness after ARCR, similar to Kim et al. [11]. However, IGHL was not released, which can be an invasive procedure in patients who underwent ARCR with no preoperative stiffness. There were no significant differences between the groups in all ROM and all functional scores at any of the assessed time points.

Although capsulectomy is considered to be an effective procedure for patients with preoperative stiffness [40, 44, 45], there is no consensus in the literature regarding the optimal extent of a glenohumeral ligament release. Bowen et al., in their cadaveric study, showed that releasing the SGHL, the MGHL, the RI, and the CHL resulted in increased ER of the shoulder joint. Releasing the anteroinferior glenohumeral ligament and the anteroinferior capsule increased elevation, and releasing the posterior-superior capsule increased IR [46]. Further comparative studies are needed according to the optimal extent of a glenohumeral ligament release, including with or without the CHL release.

In this study, all patients underwent ARCR using the suture-bridge technique. This technique revealed a superior contact area and contact pressure for the footprint of the rotator cuff stump [29, 47]. Because the technique also demonstrated excellent fixation, it has been widely used for ARCR. The main cause of retear in the suture-bridge technique has been suggested to be a medial cuff failure caused by over-tensioning the medial row [29]. The use of the knotless technique in the medial row is still controversial. It has been reported that the knotless suture-bridge technique in medial row anchors reduced retears at the musculotendinous junction and that this technique could be used to avoid necrosis of tissue caused by knot tying at the medial row anchors [48]. In this study, all patients underwent the use of the knotless suture-bridge technique in the medial row. Moreover, the major factors for retears after ARCR are said to be tissue quality and tear size. Tear size is especially associated with retears, and the retear rate of large-to-massive postoperative retears is high [29]. In this study, tear size of 80 patients (93%) were small-to-medium tears. It is difficult to know the precise reason, these might be the reasons why our study had lower retear rate compared past studies.

Our study has several limitations. First, the study design was retrospective. Second, the number of enrolled patients was relatively small. Third, the mean follow-up period of 15.6 months was relatively short. Finally, the MGHL has the greatest variation in its shape and size among all the ligaments of the shoulder joint [18, 49, 50]. The common variations of MGHL include a sublabral foramen, cord-like MGHL, and the Buford complex [49, 50]. The incidence rate is reported to range from 8 to 12% for the sublabral foramen, 1.5–5% for the Buford complex, and 19–23% for the cord-like MGHL. We could not evaluate the variation of the MGHL in this study.

## Conclusions

The preemptive MGHL release in ARCR does not significantly change the overall clinical outcomes because there were no significant differences in all ROM and all functional scores at any of the assessed time points between the groups. Moreover, there were also no significant difference in the healing failure rate and postoperative stiffness between the groups. ARCR with preemptive MGHL release could not be an effective method to reduce postoperative stiffness.

## Data Availability

The datasets used and/or analysed during the current study are available from the corresponding author on reasonable request.

## List of abbreviations

MGHL: middle glenohumeral ligament release
ARCR: arthroscopic rotator cuff repair
ROM: range of motion
CHL: coracohumeral ligament
RI: rotator interval
ER: external rotation
IR: internal rotation
MRI: magnetic resonance imaging
JOA score: Japanese Orthopedic Association Shoulder Score
UCLA score: the University of California, Los Angeles Score
